# Prognostic indicators of adverse renal outcome and death in acute kidney injury hospital survivors

**DOI:** 10.15171/jrip.2016.14

**Published:** 2016-06-01

**Authors:** Aida Hamzić-Mehmedbašić, Senija Rašić, Merima Balavac, Damir Rebić, Marina Delić-Šarac, Azra Durak-Nalbantić

**Affiliations:** ^1^Clinic of Nephrology, University Clinical Center Sarajevo, Bolnička 25, 71 000, Sarajevo, Bosnia and Herzegovina; ^2^Bournemouth University, Christchurch House C208, Talbot Campus, Fern Barrow, Poole, BH12 5BB, United Kingdom; ^3^Institute of Clinical Immunology, University Clinical Center Sarajevo, Bolnička 25, 71 000, Sarajevo, Bosnia and Herzegovina; ^4^Clinic for Heart Disease and Rheumatism, University Clinical Center Sarajevo, Bolnička 25, 71 000, Sarajevo, Bosnia and Herzegovina

**Keywords:** Acute kidney injury, Prognostic factors, Post-discharge mortality, Adverse renal outcome

## Abstract

**Introduction:** Data regarding prognostic factors of post-discharge mortality and adverse renal function outcome in acute kidney injury (AKI) hospital survivors are scarce and controversial.

**Objectives:** We aimed to identify predictors of post-discharge mortality and adverse renal function outcome in AKI hospital survivors.

**Patients and Methods:** The study group consisted of 84 AKI hospital survivors admitted to the tertiary medical center during 2-year period. Baseline clinical parameters, with renal outcome 3 months after discharge and 6-month mortality were evaluated. According survival and renal function outcome, patients were divided into two groups.

**Results:** Patients who did not recover renal function were statistically significantly older (*P* < 0.007) with higher Charlson comorbidity index (CCI) score (*P* < 0.000) and more likely to have anuria and oliguria (*P* = 0.008) compared to those with recovery. Deceased AKI patients were statistically significantly older (*P* < 0.000), with higher CCI score (*P* < 0.000), greater prevalence of sepsis (*P* =0.004), higher levels of C-reactive protein (CRP) (*P* < 0.017) and ferritin (*P* < 0.051) and lower concentrations of albumin (*P*<0.01) compared to survivors. By multivariate analysis, independent predictors of adverse renal outcome were female gender (*P* =0.033), increasing CCI (*P* =0.000), presence of pre-existing chronic kidney disease (*P* =0.000) and diabetes mellitus (*P* =0.019) as well as acute decompensated heart failure (ADHF) (*P* =0.032), while protective factor for renal function outcome was higher urine output (*P* =0.009). Independent predictors of post-discharge mortality were female gender (*P* =0.04), higher CCI score (*P* =0.001) and sepsis (*P* =0.034).

**Conclusion:** Female AKI hospital survivors with increasing burden of comorbidities, diagnosis of sepsis and ADHF seem to be at high-risk for poor post-discharge outcome.

**Implication for health policy/practice/research/medical education:**

Female acute kidney injury (AKI) hospital survivors with increasing burden of comorbidities, diagnosis of sepsis and acute decompensated heart failure (ADHF) seem to be at high-risk for poor postdischarge outcome. Close monitoring of high-risk hospital AKI survivors after discharge should be done, preferably by a nephrologists, aiming to reduce mortality and prevent adverse outcome of kidney function in this patient population.

## Introduction

Acute kidney injury (AKI) is associated with significantly increased in-hospital and long-term morbidity and mortality (1)*.* The identification of risk factors associated with AKI and its poor prognosis is required, so that preventive and early diagnosis measures can be taken, aiming to reduce mortality of AKI patients. Most published studies have focused on prediction of in-hospital mortality in AKI patients (2-4). Furthermore, studies examining long-term postdischarge mortality have focused primarily on critically ill patients with AKI that requires dialysis (5).

Acquisition of a better understanding of AKI hospital survivors, who are at increased risk of progressive chronic kidney disease (CKD), and death, is of vital public health importance. Currently there is lack of consensus for defining renal recovery. Recovery of renal function after AKI has been primarily described in patients who required renal replacement therapy (RRT) and largely defined as subsequent independence from dialysis at hospital discharge. Recently, it is established that even milder forms of AKI not requiring dialysis are associated with both short-term and long-term mortality (6). Recognition that AKI survivors are at high risk of progressive CKD spurred the Kidney Disease Improving Global Outcomes (KDIGO) AKI guidelines to recommend that the kidney function should be evaluated 3 months after an AKI episode to establish the presence and extent of CKD (7).

To our best knowledge, data regarding predictors of adverse renal function outcome, defined using 3 months time frame for AKI recovery, are scarce. Furthermore, prognostic factors associated with post-discharge mortality of AKI hospital survivors are controversial. Some authors (8)have shown that the most important risk factors for post-discharge mortality are patients’ characteristics, especially advanced age, male gender and presence of comorbidities. However, other authors (9) have associated AKI characteristics, in particular AKI severity, with post-discharge mortality.

## Objectives

The main objective of this investigation was to identify significant predictors of postdischarge mortality and adverse renal function outcome (defined using 3 months time frame as recommended according KDIGO guidelines) in AKI hospital survivors.

## Patients and Methods

### Study design and patients

This prospective study was carried out at Clinic of Nephrology in the University Clinical Center Sarajevo from July 1, 2012 to December 31, 2014. Patients included in this study were adult (age ≥18 years) hospital survivors with diagnosis of AKI and hospital stay longer than 24 hours. Patients with prior kidney transplant, end-stage kidney disease (ESRD) and initiation of RRT before hospital admission were excluded. All participants signed the informed consent.

AKI patients who met inclusion criteria have been followed up for 6 months after hospital discharge. According outcome of renal function 3 months following discharge, patients were divided into two groups: those who recover renal function and those who fail to achieve renal function recovery. According survival 6 months after discharge, the patients were divided into two groups, survivors and non-survivors. These divisions of patients were done in order to investigate differences in relation to all studied parameters.

### Data collection

Clinical, demographic and laboratory features at admission were studied. Age and gender of patients, length of hospital stay, urinary volume, severity of AKI, different treatment modalities (conservative treatment or RRT) and systemic comorbidities were recorded. We included both the Charlson comorbidity index (CCI) score and individual components of score in our study including pre-existing CKD. CCI score was calculated using standard algorithm proposed by Quan et al (10). Laboratory tests included levels of serum creatinine (SCr), hemoglobin (Hgb), erythrocyte sedimentation rate (ESR), C-reactive protein (CRP), white blood cell counts (WBC), serum albumin, ferritin, cholesterol and uric acid.

### Definition and criteria of AKI

AKI was defined as an abrupt (within 48 hours) absolute increase in SCr of at least 26.5 μmol/L (0.3 mg/dL) or by a percentage increase in SCr ≥50% from baseline according to the Acute Kidney Injury Network (AKIN) criteria (11). The severity of AKI was defined by the AKIN staging criteria(11) as follows: stage 1, SCr increase to 1.5–2 fold from baseline; stage 2, SCr increase >2–3 fold from baseline; and stage 3, SCr increase >3.0 fold from baseline or SCr ≥354 μmol/l (≥4.0 mg/dl) with an acute increase of at least 44 μmol/l (0.5 mg/dL) or need for RRT. When pre-admission serum creatinine was unavailable, it was estimated by the Modification of Diet in Renal Disease (MDRD) equation, with the assumption of a near lower limit of normal glomerular filtration rate (GFR) of 75 mLl/min/1.73 m^2^, as recommended by the Acute Dialysis Quality Initiative (ADQI) Working Group (12).

Oliguria was considered to be present when the urinary volume was less than 400 mL/day and anuria if the urinary volume was less than 100 mL/day.

### Outcome of AKI

The primary outcome of AKI patients was in-hospital mortality, while the secondary outcome was 6-month mortality. The outcome of renal function was defined 3 months after discharge by the values of estimated GFR (eGFR) as recovered (eGFR >60 ml/min/1.73 m^2^) or non-recovered (eGFR <60 ml/min/1.73 m^2^). This definition of renal function outcome refers only to AKI patients without pre-existing CKD. AKI patients with presumed CKD were considered to have renal function recovery if SCr concentrations fell to the baseline 3 months after discharge. Non-recovery of kidney function was defined if SCr remained above the baseline. A 3 months time frame for recovery was used based on the KDIGO AKI guidelines (7). Informations related to renal function outcome and survival were obtained from the family doctor or nephrologists in our medical centre and taken from the death register.

### Ethical issues

The research followed the tenets of the Declaration of Helsinki. Informed consent was obtained and the research was approved by the Ethics Committee of the University Clinical Center Sarajevo (0207-27144).

### Statistical analysis

Normally or near normally distributed continuous variables were reported as means with standard deviations and compared by Student’s *t* test. Non-normally distributed continuous data were reported as medians with interquartile ranges and compared using Mann-Whitney U test. When outcome variable was binary or categorical, proportion between two or more groups was assessed by chi-square test or Fischer exact test when there was sparse data. Finally, logistic regression analysis was used to assess the determinants of renal function outcome and mortality. Model fit was assessed by the goodness-of-fit tests. Data were presented as odds ratios (ORs) and marginal effects with 95% CI. *P* values less than 0.05 were considered statistically significant. Statistical analysis was realized using SPSS software (version 16).

## Results

Of the total of 1231 patients admitted in the study period, AKI occurred in 96 cases (7.8 %). The cohort of 84 AKI survivors (41 females and 43 males) with mean age of 73.5 years met inclusion criteria. Majority of patients had great AKI severity (Stage 3 in 78.5% of cases), with prevalence of pre-existing CKD in 54.8% of AKI patients. Comorbidities were present in 77.4% of cases with mean CCI score 6.4± 3.05. The most common causes of AKI were acute interstitial nephritis (AIN) and acute decompensated heart failure (ADHF) in 16.7% and 15.5% of patients, respectively, followed by acute gastroenterocolitis (13.1%) and sepsis (12%), while the remaining etiologies participated with less than 10.5%. The majority of patients (78.6%) received conservative treatment while 21.4% of them underwent RRT.

Renal function recovery was recorded in 48.8% of AKI patients three months after discharge. Prevalence of intra-hospital and 6-month mortality was 12.5% and 45.2%, respectively. A comparison of demographic, laboratory and clinical data between patients who recovered renal function and those who did not is summarized in Table 1. Patients who did not recover renal function were statistically significantly older (*P*<0.007), with higher CCI score (*P*<0.000), higher values of SCr at discharge (*P*<0.018) as well 3 months after discharge (*P*<0.000) in comparison to patients who recovered kidney function. In addition, anuria and oliguria were significantly more common in the group of patients with renal function non-recovery (*P*=0.008).

**Table 1:** Comaparison of characteristics between AKI patients according renal function outcome

	**Recovery of renal function (n=43)**	**Non-recovery of renal function (n=41)**	***P***
Sex			0.192
Male (n, %)	25 (58.14)	18 (41.86)	
Female (n, %)	18 (43.9)	23 (56.1)	
Age (years)	69 (55-78)	78 (69-82)	<0.007*
Length of hospital stay (days)	16.5 (10-23)	15 (10-24)	0.567
Hgb (g/L)	124.7±25.2	116.4 ± 21.06	0.105
WBC (x10^9^/L)	12.8 (10.1-14.5)	11.5 (7.6-14)	0.316
ESR	69.5 (24-97.5)	57 (41-115)	0.954
CRP (mg/L)	63.1 (27.2-31.6)	64.7 (28.5-140.7)	0.639
Ferritin (ng/mL)	409.1 (160.7-534.7)	360.6 (220.8-757.5)	0.597
Albumin (g/L)	29.4 ± 6.2	27.7 ± 6.5	0.25
Cholesterol (mmol/L)	4.3 ± 1.4	3.8 ± 1.5	0.192
Uric acid (μmol/L)	507 ± 191.9	616.03 ± 272.3	0.062
SCr levels (μmol/L) at admission	462 (363-610)	421 (246-660)	0.407
SCr levels (μmol/L) at discharge	130 (93-198)	175 (116-132)	<0.018*
SCr levels (μmol/L) 3 months after discharge	98 (79-174)	172 (118-317)	<0.000*
Diuresis			0.008*
Anuria (n, %)	1 (16.67)	5 (83.33)	
Oliguria (n, %)	2 (18.18)	9 (81.82)	
Diuresis >400 mL (n, %)	40 (59.7)	27 (40.3)	
AKI etiology			
AIN (n, %)	9 (64.29)	5 (35.71)	0.283
ADHF (n, %)	4 (30.77)	9 (69.23)	0.096
Gastroenterocolitis (n, %)	7 (63.64)	4 (36.36)	0.521
Sepsis (n, %)	3 (30)	7 (70)	0.19
Pre-existing CKD			0.120
Yes (n, %)	20 (43.48)	26 (56.52)	
No (n, %)	23 (60.53)	15 (39.47)	
Diabetes mellitus			0.190
Yes (n, %)	15 (62.5)	9 (37.5)	
No (n, %)	28 (46.67)	32 (53.33)	
Hypertension			0.871
Yes (n, %)	15 (50)	15 (50)	
No (n, %)	28 (51.85)	26 (48.15)	
CCI score	5.1 ± 2.8	7.8 ± 0.4	< 0.000*
Treatment			0.239
RRT (n, %)	7 (38.89)	11 (61.11)	
Conservative (n, %)	36 (54.45)	30 (55.55)	

Abbreviations: AKI, acute kidney injury; n, number; Hgb, hemoglobin; WBC, white blood cell counts; ESR, erythrocyte sedimentation rate; CRP, C-reactive protein; SCr, serum creatinine; AIN, acute interstitial nephritis; ADHF, acute decompensated heart failure; CKD, chronic kidney disease; CCI, Charlson comorbidity index; RRT, renal replacement therapy. Data are presented as median and interquartile range or as means and standard deviation, * *P*<0.05.

Table 2 , illastrated the comparison of characteristics between AKI patients according renal function outcome. A comparison of characteristics of surviving and deceased AKI patients is shown in Table 2. Deceased AKI patients were statistically significantly older (*P*<0.000), with higher prevalence of septic etiology (*P*=0.004), higher CCI score (*P*<0.000), lower values of serum albumin (*P*<0.01), higher concentrations of CRP (*P*<0.017) and ferritin (*P*<0.051) as well as higher values of SCr at discharge (*P*<0.022) and 3 months following discharge (*P*<0.000) with lower eGRF 3 months following discharge (*P*<0.000) compared to survivors.

**Table 2:** Comparison between characteristics of AKI patients according survival

	**Survivors (n=46)**	**Non-survivors (n=38)**	***P***
Sex			0.843
Male (n, %)	24 (55.81)/	19 (44.19)	
Female (n, %)	22 (53.66)	19 (46.34)	
Age (years)	67.5 (53-78)	78 (70-80)	<0.000*
Length of hospital stay (days)	16.5 (12-23)	16 (9-13)	0.636
Hgb (g/L)	123. ±23.5	117.7±23.4	0.312
WBC (x10^9^/L)	11.78 (8.9-3.8)	13 (7.6-15)	0.361
ESR	67 (27-95)	65 (39-112.5)	0.946
CRP (mg/L)	48.9 (22.4-100.2)	95.8 (58.7-141.5)	<0.017*
Ferritin (ng/mL)	278.3 (135.7- 483.5)	628.5 (299.1- 837)	<0.051*
Albumin (g/L)	30.2±6.5	26.4±5.7	<0.01*
Cholesterol (mmol/L)	4.3±1.3	3.9±1.6	0.265
Urine acid (μmol/L)	517.3±198.6	621.1±280.2	0.082
SCr levels (µmol/L) at admission	420.5 (295-446)	469 (337-663)	0.305
SCr levels (µmol/L) at discharge	123.5 (98-181)	175.5 (111-321)	<0.022*
SCr levels (µmol/L) 3 months after discharge	118.5 (81-174)	173.5 (116-321)	<0.000*
eGFR 3 months after discharge	56.9±29.6	32.2 ±18.7	< 0.000*
Diuresis			<0.103
Anuria‏ (n‏, %)	1 (16.67)	5 (83.33)	
Oliguria‏ (n‏, %)	5 (45.45)	6 (54.55)	
Diuresis‏ >400mL‏ (n‏, %)	40 (59.70)	27 40 (40.30)	
AKI etiology			
AIN‏ (n‏, %)	6 (42.86)	8 (57.14)	0.327
ADHF‏ (n‏, %)	9 (69.23)	4 (30.77)	0.202
Gastroenterocolitis‏ (n‏, %)	9 (69.23)	4 (30.77)	0.1
Sepsis‏ (n‏, %)	1 (10)	9 (90)	0.004*
Pre-existing CKD			0.216
Yes‏ (n‏, %)	18 (47.37)	20 (52.63)	
No‏ (n‏, %)	28 (60.87)	18 (39.13)	
Diabetes			0.677
Yes‏ (n‏, %)	14 (58.33%)/	10 (41.67%)/	
No‏ (n‏, %)	32 (53.33%)	28 (46.67%)	
Hypertension			0.845
Yes‏ (n‏, %)	16 (53.33%)/	14 (46.67%)/	
No‏ (n‏, %)	30 (55.55)	24 (44.55%)	
CCI score	4.5±0.3	8.7±0.3	< 0.000*
Treatment			0.647
RRT‏ (n‏, %)	9 (50%)	9 (50%)	
Conservative‏ (n‏, %)	37 (56.1%)	29 (43.9)	

Abbreviations: AKI, acute kidney injury; n, number, Hgb, hemoglobin; WBC, white blood cell counts; ESR, erythrocyte sedimentation rate; CRP, C-reactive protein; SCr, serum creatinine; eGFR, estimated glomerular filtration rate; AIN, acute interstitial nephritis; ADHF, acute decompensated heart failure; CKD, chronic kidney disease; CCI, Charlson comorbidity index; RRT, renal replacement therapy. Data are presented as median and interquartile range or as means and standard deviation, * P<0.05.

Table 2, revealed the comparison between characteristics of AKI patients according to survival.

To observe factors which determine probability that patient recovers renal function and probability that patient dies, set of logit estimates is performed. *A priori* selected variables included age, gender, length of hospital stay, urine output, AKIN stage, AIN, gastroenterocolitis, ADHF, sepsis, pre-existing CKD, hypertension, diabetes mellitus, CCI score and type of treatment. Multivariate regression analysis (Table 3) showed that significant independent predictors of adverse renal function outcome were female gender (*P*=0.033), pre-existing CKD (*P*=0.000), higher CCI (*P*=0.000), diabetes mellitus (*P*=0.019) and ADHF (*P*=0.032). Estimated results in Table 3 also suggested that probability that female patients would recover renal function was 15.6% lower compared to male patients. Patients with higher CCI score had also less chance for renal function recovery. For example, one unit increase in CCI score would reduce probability of recovery by 7.7%. Furthermore, patients with underlying CKD had 35.6% less probability to recover renal function than patients without pre-existing CKD, while diabetes mellitus and ADHF reduced chance of recovery by 25% and 25.6%, respectively. Unlike these results, higher urine output proved to be independent factor of recovery of renal function (*P*=0.009). Patients with higher urine output had 16.8% higher probability to recover kidney function.

**Table 3:** Significant independent predictors of postdischarge renal function outcome and mortality in AKI hospital survivors

**Variables**	**OR**	**95% CI**	***P***	**Margins (predicted probabilities)**
**Renal function outcome**				
Female	0.273	‏0.08-0.9	‏0.033*	‏-0.156
Urine output	4.065	‏1.41-11.7	‏0.009*	‏0.168
ADHF	0.119	‏0.01-0.83	‏0.032*	‏-0.256
Pre-existing CKD	0.051	‏0.01-0.21	‏0.000*	‏-0.356
Diabetes mellitus	0.125	‏0.02-0.71	‏0.019*	‏-0.25
CCI score	0.524	‏0.36-0.75	‏0.000*	‏-0.077
**Mortality**				
Female	3.157	0.13-‏6.17	‏0.04*	‏0.163
Sepsis	7.115	‏0.536 - 13.69	‏0.034*	‏0.368
CCI score	2.181	‏0.84-3.51	‏0.001*	‏0.113

Abbreviations‏: OR‏, Odds‏ ratio‏; AKI‏, acute‏ kidney‏ injury‏; ADHF‏, acute‏ decompensated‏ heart‏ failure‏; CKD‏, chronic‏ kidney‏ disease‏; CCI‏, Charlson‏ comorbidity‏ index‏. ‏*P<0.05.

Significant independent predictors of postdischarge renal function outcome and mortality in AKI hospital survivors was shown in Table 3.

According to estimated results in Table 3 female gender (*P*=0.04), higher CCI score (*P*=0.001), and sepsis (*P*=0.034) were significant independent risk factors for death. Females had 16.3% higher probability to die compared to males. Diagnosis of pre-existing CKD and sepsis increased probability of mortality in AKI patients by 19.3% and 36.8%, respectively. In addition, the probability of death enhanced by 11.3% with each rise of CCI score by one unit.

**Figure 1:**
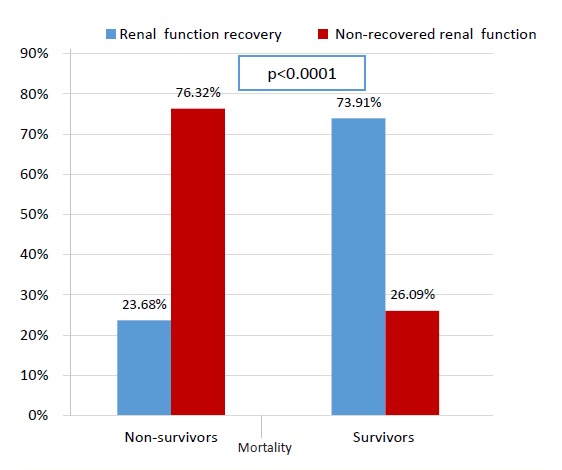
Comparison of different renal function outcome between surviving and deceased acute kidney injury patients

Finally, Figure 1 shows that non-recovery of renal function was found in statistically significantly greater proportion of non-survivors compared to survivors (76.32% versus 26.09%, *P*<0.0001).

Comparison of different renal function outcome between surviving and deceased AKI patients was shown in Figure 1.

## Discussion

Present study was conducted in order to identify significant predictors of adverse renal function outcome and death in AKI hospital survivors. AKI is common in the clinic, occurring in 8% of all in-hospital patients and in approximately 50% of patients in the Intensive Care Units (13)*.* The occurrence of AKI in our referral hospital centre (7.8%) is similar as previously reported (13). Of the 84 AKI patients in our study group, majority of them were older (median age 73.5 years) with great severity of AKI (AKIN stage 3 in 78.6% of cases) and high burden of comorbidities (comorbid conditions were present in 77.4% of cases with mean CCI score 6.4 ± 3.05). Prevalence of pre-existing CKD was rather high too (54.8%). As previously reported (14), comorbidities are common among elderly patients and both CKD and AKI are often associated.In accordance with our results, great proportion of AKI patients, classified as stage three, was found in earlier study (15) which evaluated AKI patients followed by nephrologists.

Evaluation of kidney function outcome in our study was done three months after an AKI episode according KDIGO AKI guidelines (7). Renal function recovery was established in 48.8% of AKI patients, which is similar to proportion of patients with renal function recovery in the studies of Pereira and Yang with their associates (50% and 58.6%, respectively) (15,16).

Identification of predictors of adverse renal function outcome in AKI survivors has importance in order to timely implement preventive measures for high-risk patients (2). Similarly to recent findings of Harel et al (17), higher CCI score was an independent predictor of renal function non-recovery in our study, implying that CCI score could be beneficial, not only for predicting risk for death from comorbidities, but for predicting adverse renal function outcome of AKI patients. Furthermore, by multivariate analysis, pre-existing CKD was proved to be an independent prognostic indicator of poor renal function outcome in present study. Recent studies have also demonstrated the association between pre-existing CKD and the progression of kidney disease (17,18). The impact of even modest acute insult on already compromised kidneys may be devastating. Although the biological mechanism linking CKD progression after AKI has not fully been elucidated, it has been postulated that the combination of acute endothelial damage leading to vascular dropout, and nephron loss followed by glomerular hypertrophy with the development of fibrosis may all play a role (19).

Diabetes mellitus is the most important contributor to the growing burden of ESRD, and patients with diabetes mellitus are at a greater risk of requiring hospitalizations and experiencing AKI. Recent findings (20) indicate that, in the setting of diabetes, AKI increases the risk of advanced CKD by over three-fold. Our study identified the presence of diabetes mellitus, as significant independent risk factor for adverse renal function outcome in AKI survivors, which is in accordance with the results of other authors (6). Another independent predictor of deterioration of renal function in our AKI patients was diagnosis of ADHF. Pannu et al (6) and Gao et al (21) also confirmed that patients with heart failure were less likely to recover renal function. These findings are not unexpected since recent evidence implies that more than 70% of patients hospitalized for ADHF will experience acute worsening of renal function, which is associated with significantly poor outcomes (22). Furthermore, in accordance with recent study (21), our results showed less chance of kidney function recovery in anuric and oliguric AKI patients.

Despite significant advances in supportive care, the mortality rate of AKI has not significantly improved over the past several decades. On a contrary, as our study and Gammelager et al (23) reported, the rate of in-hospital mortality seemed to be growing after hospital discharge. Present study showed that hypoalbuminemia as well as increased CRP and ferritin were significantly more common in deceased in comparison to survived AKI patients. A poor prognosis of AKI patients who have hypoalbuminemia was described in previous research (24). It seemed that patients had worse nutritional status with suppressed synthesis of albumin as response to inflammatory condition (24). Those observations were confirmed in recent study which revealed that the presence of low caloric intake and higher CRP levels were significantly associated with risk of death in AKI patients (25).

By multivariate analysis, significant independent predictors of postdischarge mortality in the cohort of our AKI hospital survivors were female gender, higher CCI score and diagnosis of sepsis. Higher mortality rate was earlier detected in aged females due to the more prevalent hypervolemia and delayed initiation of dialysis (26). In accordance with our findings, higher CCI score was found to be predictive of post-AKI mortality in recent study (17).

Distinctive clinical features of AKI of septic etiology have been earlier elucidated, implying that unique pathogenesis contributes to the development of septic AKI (27). The mortality of sepsis induced AKI is more than 70% (28). Present study confirmed that septic cause of AKI was statistically significantly more common in non-survivors compared to survivors. Septic AKI has been independently associated with an in­creased risk for death in published studies (28,29) as well as in our research.

Analysis of kidney function outcome and post-discharge mortality in our study revealed that non-recovery of renal function was statistically significantly more common in deceased AKI patients. The association between kidney function non-recovery and mortality was earlier reported, but its magnitude depended on both recovery definition and the timing of assessment (30), highlighting that a standardized definition for renal recovery in AKI is needed in future investigations.

## Conclusion

Female gender, higher CCI score, presence of presumed CKD, diabetes mellitus and ADHF are significant high-risk prognostic factors for poor outcome of renal function in AKI patients, while protective factor for kidney function outcome is increased urine output. Furthermore, female AKI survivors with high burden of comorbidities and diagnosis of sepsis are at high risk for postdischarge death. Close monitoring of high-risk hospital AKI survivors after discharge should be done, preferably by a nephrologists, aiming to reduce mortality and prevent adverse outcome of kidney function in these patients population.

## Limitations of the study

The potential limitation of present study is that it consists of relatively small sample size. However, this sample size reflects the two-year occurrence of AKI in our tertiary medical center. We suppose to perform larger multicenter study.

## Authors’ contribution

AHM and SR collected the data and wrote the manuscript. MB analyzed the data. DR and MDŠ contributed to data collection, analysis and preparation of the manuscript. ADN helped with the study design and data collection.

## Conflicts of interest

The authors declare no conflict of interests.

## Ethical considerations

Ethical issues (including plagiarism, data fabrication, double publication) have been completely observed by the authors.

## Funding/Support

None.
